# The Science of Aging: Understanding Phenolic and Flavor Compounds and Their Influence on Alcoholic Beverages Aged with Alternative Woods

**DOI:** 10.3390/foods14152739

**Published:** 2025-08-05

**Authors:** Tainá Francisca Cordeiro de Souza, Bruna Melo Miranda, Julio Cesar Colivet Briceno, Joaquín Gómez-Estaca, Flávio Alves da Silva

**Affiliations:** 1 Escola de Agronomia e Engenharia de Alimentos, Universidade Federal de Goiás, Goiânia 74690-900, GO, Brazil; tainafcs@hotmail.com (T.F.C.d.S.); bruna.melo.miranda@gmail.com (B.M.M.); juliocolivet@gmail.com (J.C.C.B.); flaviocamp@ufg.br (F.A.d.S.); 2 Institute of Food Science, Technology and Nutrition (ICTAN-CSIC), C/José Antonio Novais 6, 28040 Madrid, Spain

**Keywords:** aging of alcoholic beverages, alternative woods, phenolic compounds, sensory profile, innovation in maturation

## Abstract

Aging in wooden barrels is a proven technique that enhances the sensory complexity of alcoholic beverages by promoting the extraction of volatile and phenolic compounds. While oak has been traditionally used, there is a growing interest in exploring alternative wood species that can impart distinct sensory characteristics and promote innovative maturation processes. This review examines the impact of alternative woods on the aging of beverages, such as wine, cachaça, tequila, and beer, focusing on their influence on aroma, flavor, color, and chemical composition. A bibliometric analysis highlights the increasing scientific attention toward wood diversification and emerging aging technologies, including ultrasound and micro-oxygenation, which accelerate maturation while preserving sensory complexity. The role of toasting techniques in modulating the release of phenolic and volatile compounds is also discussed, emphasizing their contribution to unique sensory profiles. Additionally, regulatory aspects and sustainability considerations are explored, suggesting that alternative woods can expand flavor possibilities while supporting environmentally sustainable practices. This review underscores the potential of non-traditional wood species to drive innovation in the aging of alcoholic beverages and provide new sensory experiences that align with evolving consumer preferences and market trends.

## 1. Introduction

The sensory properties of alcoholic beverages, including flavor, aroma, and color, are influenced by various factors, ranging from raw materials and fermentation processes to production technologies and maturation techniques. Among these factors, the use of wooden containers has been an essential element in defining the chemical and sensory profile of many beverages. Traditionally, barrels made of French and American oak are the most widely used due to their unique composition, which facilitates the extraction of various chemical compounds such as esters, lactones, and phenols. These compounds directly impact the complexity and final quality of the product [1,2,3,4,5].

Oak is valued not only for its durability and ease of processing but also for its distinct chemical properties. During the aging process, the thermal treatment of oak barrels initiates the decomposition of lignin, hemicellulose and cellulose. This degradation leads to the formation of volatile and non-volatile compounds that give the beverages characteristic smoky, woody and vanilla flavors [6,7,8,9]. The interaction between the beverage matrix and wood extractives also promotes oxidation reactions and other chemical transformations that further enhance the sensory profile. These interactions have established traditional oak aging as a quality benchmark for wines, spirits, and even specialty beers [6,10,11,12,13].

In recent years, however, there has been increasing interest in the use of alternative wood species and products made from them, such as chips or staves, as a substitute for conventional oak. This emerging trend is driven by several factors, including economic considerations, environmental concerns and legislative changes. The use of alternative woods can bring new and distinct chemical profiles to beverages, enabling innovative sensory experiences. In addition, these alternatives can mitigate the limitations associated with the availability and cost of oak while allowing for more controlled and reproducible aging processes [10,14,15]. Although a historical overview of the dominance of oak in beverage maturation is useful for context, the main focus of this review is to critically evaluate the state of the art in the use of alternative woods and compare their impact on the sensory characteristics of alcoholic beverages compared to traditional oak.

To identify the latest advances and trends in this field, we conducted a systematic literature search of several databases, including Science Direct, Scielo, and SpringerLink. Our search strategy employed a combination of English terms, such as “aged beverage” OR “beverage aging” in conjunction with “wooden”, “wood”, “barrel”, “chip”, “wood maturation”, and “oak”, specifically applied to article titles and abstracts. We limited our search to publications from the last ten years (2014–2024) and initially identified 65 studies, which were then assessed for relevance to the objectives of this review. In addition, we manually included several seminal works published prior to 2014 (e.g., 1995, 1996, 1998) that are frequently cited in historical reviews of wood aging. However, the use of specific search terms may have excluded some relevant manuscripts in which aging was addressed as a general or secondary parameter. Therefore, this review may not encompass all studies evaluating aged alcoholic beverages.

Thus, this study aims to provide a comprehensive comparison between traditional oak aging and the use of alternative wood species and products. By synthesizing the latest findings from scientific research, we seek to elucidate how different types of wood influence the chemical composition and sensory characteristics of alcoholic beverages. This analysis not only highlights the potential benefits of using alternative woods but also identifies challenges and limitations that should be addressed in future research. The goal is to provide a balanced perspective that contributes both to the advancement of scientific knowledge and to practical applications in the beverage industry.

## 2. Scientific Prospection

### 2.1. Overview

The aging of alcoholic beverages in wood has gained increasing attention in scientific research over the past decade. Studies have primarily focused on the sensory profile modifications induced by wood contact, the chemical composition of beverages aged in different types of wood, and the development of innovative maturation technologies. This growing interest underscores the relevance of exploring alternative wood species beyond the traditional oak for aging purposes.

### 2.2. Application Area

Wood aging influences the production of various alcoholic beverages, contributing to their sensory complexity and chemical stability. The most commonly aged beverages include sugarcane spirits (cachaça and rum) [16], wine [10], tequila [17], general spirits [18], and beer [13]. Cachaça, a traditional Brazilian sugarcane spirit, accounts for a considerable share of wood-aged beverages, reflecting its cultural and economic relevance in Brazil [19]. Similarly, wine aging in barrels is a well-established practice worldwide, particularly in Europe, where countries such as Spain, France, Italy, and Portugal lead production [20].

Tequila, a spirit with a protected designation of origin, undergoes specific aging processes that influence its final characteristics, making wood selection a determining factor [21]. Beer, although less commonly aged in wood compared to spirits and wine, has seen a rise in experimental aging techniques, especially among craft breweries aiming to create distinctive sensory profiles [11].

The use of alternative woods for aging is particularly important in regions where traditional oak is less accessible or where local wood species have unique properties. For example, Martínez-Gil et al. characterized tannin and low-molecular-weight phenols in Colombian oak as an alternative cooperage material [20]. This aspect emphasizes the opportunity for innovation in the beverage industry by aligning scientific research with market demand for diversified and distinctive products.

## 3. Technological Prospection

### 3.1. The Origins and Evolution of Barrel Aging Techniques

The use of wooden barrels in alcoholic beverage production originally served a utilitarian purpose—facilitating storage, transport, and preservation due to their robustness and ease of handling. Over time, however, empirical observations revealed that the intrinsic properties of wood could actively contribute to the sensory profile of the liquid, as contact with the staves imparts desirable compounds such as esters, lactones, and phenols [22,23,24]. This realization transformed barrels from passive containers into essential maturation tools, prompting innovations in cooperation. Advances in wood selection, seasoning, staves, and toasting protocols were developed to modulate the release kinetics of key extractives, thereby allowing producers to deliberately shape flavor, aroma, and mouthfeel. Such refinements elevated barrel aging from a mere storage step to a controlled, artful process [25,26,27].

These technological improvements not only standardized the quality of barrel-aged products but also paved the way for further experimentation with wood treatments. As modern practices evolved, the focus shifted towards optimizing the balance between efficient extraction and the preservation of the beverage’s inherent characteristics. This historical and technical progress underscores the shift from using barrels solely for storage to employing them as dynamic agents in the aging process, thereby contributing directly to the sensory quality of the final product. The following sections describe the types of wood used and the specific mechanisms involved in the aging process, while discussing how technological innovations continue to enhance beverage quality.

### 3.2. Influence of the Physicochemical Properties and Chemical Composition of Wood on the Aging of Alcoholic Beverages

The wood used for the maturation of alcoholic beverages plays a decisive role in shaping the sensory profile of the final product. This effect results from the intrinsic physicochemical properties of its cellular structure, such as density, hardness and porosity, and the chemical composition of its constituents. In general, the density and hardness of the wood are related to its mechanical resistance, while the porosity—defined as the volume of voids within the structure—controls the diffusion of liquids and gases through the wood matrix. This diffusion enables a cascade of oxidation, hydrolysis, esterification and polymerization reactions involving phenolic compounds that progressively change the aroma, color, and texture of the beverage. Structural differences between wood species have a direct influence on the rate and extent of transfer of aromatic compounds to the aging liquid [27,28].

Thus, wood should not be regarded as a passive container during aging; rather, it acts as an active medium in the extraction and transformation of compounds. The physical properties of wood—particularly density, hardness, and porosity—create a microenvironment within barrels that modulates the gradual release of extractable substances. While density affects liquid retention and the rate of evaporation (commonly referred to as the “angel’s share”), porosity facilitates interactions between the beverage and wood-derived precursors. These interactions are essential for the release of compounds, such as ellagitannins, tannins, lactones, and phenols, which originate from the wood structure itself and from subsequent reactions induced by barrel toasting and extended contact with the aging liquid [29].

The chemical transformations that occur during aging can be interpreted as a sequential and synergistic array of reactions. Initially, direct extraction of compounds occurs as the beverage penetrates the wood’s porous matrix. Hydrolytic reactions then promote the release of aromatic precursors, which, in the presence of ethanol and oxygen, undergo oxidation processes that generate lower-molecular-weight products and enhance the beverage’s sensory complexity. Furthermore, barrel toasting induces the caramelization of naturally occurring sugars in the wood, leading to the formation of volatile compounds such as furfural, which serves as a marker for toast level and aging intensity [30].

Accordingly, the integration between the physicochemical properties of wood and the chemical reactions that they promote is fundamental to the development of the aromas, flavors, and colors that characterize aged beverages. The chemical composition of wood—particularly its content of hemicellulose (25–35%), lignin (15–30%), and cellulose (40–50%)—not only defines its structural framework but also determines its extraction potential. Although cellulose is the primary structural component and relatively inert, it provides mechanical support to the barrel. Hemicellulose and lignin, in contrast, are more chemically reactive and capable of releasing a wide range of compounds over time, including sugars, phenolic acids, and key aroma and flavor precursors [31,32].

Lignin contributes in particular to the softness and texture of the beverage and helps to stabilize the pigments and prolong the color stability of the aged product. Its degradation releases phenolic aldehydes such as vanillin, syringaldehyde, coniferaldehyde and sinapaldehyde, which are responsible for vanilla, spicy and woody notes [33]. Tannins, especially those originating from lignin, are influenced by both the wood species and the processing conditions. These compounds improve the structural complexity of the beverage, contribute to astringency and body, but also act as antioxidants and color stabilizers, supporting the development of more nuanced aromas during aging [9,34,35,36,37].

The hydrolysis of hemicellulose releases pentoses and hexoses, which upon degradation form furans (e.g., furfural and 5-hydroxymethylfurfural) and pyrans, responsible for caramel, honey, and dried fruit aromas [38]. Together, these variables endow wood with the ability to modulate the evolution of aged products, promoting a synergistic interplay between extractive mechanisms and chemical transformations throughout the maturation process.

#### 3.2.1. Oak

Historically and contemporarily, oak has been the preferred material for barrel making due to a combination of factors ranging from its cellular structure to the chemical composition of its constituents—crucial factors in shaping the sensory profile of aged beverages. Oak wood, in particular, exhibits controlled porosity, which enables gradual micro-oxygenation of the beverage. This process is essential for the evolution of flavors and aromas during maturation [28]. Micro-oxygenation regulates oxidative and esterification reactions, which transform wood-derived compounds and contribute to the formation of complex aromatic profiles [39].

American white oak (*Quercus alba*) is characterized by its higher porosity and larger vessel diameters, which facilitate a rapid and intensive extraction of aromatic compounds, such as vanillin, responsible for vanilla notes, and precursors, which develop into caramel-like aromas during oxidation and soften the tannic profile. This feature makes American oak particularly suitable for products aiming at a more vibrant aromatic profile with less pronounced astringency. The higher porosity of *Q. alba* promotes the release of lactones, such as β-methyl-γ-octalactone, which impart coconut and vanilla notes, in addition to reducing the harshness of tannins and contributing to a rounder and more accessible sensory character [40].

Conversely, European oaks (*Quercus robur* and *Quercus petraea*), with denser structures and a greater presence of tyloses, allow for a more gradual release of phenolic compounds. This controlled extraction favors the development of complex aromatic profiles, with a higher persistence of tannins and formation of volatile compounds reminiscent of spices and dried fruits [41,42,43].

Oak wood is rich in tannins, lignins, lactones, and vanillin [9,34,35,36,37]. During the aging process, volatile components are intensified through mechanisms such as water evaporation, gradual compound release from the wood matrix, and the formation of new esters [44,45]. These mechanisms yield beverages with integrated and harmonious notes, where the interaction between tannins, lactones, and phenolic compounds contributes to a refined sensory balance. These characteristics have established oak as a reference material in the industry [3,4,5,7,8,9,11] but have also encouraged research into alternative wood species that can be used individually or in combination with oak to broaden the sensory spectrum of aged beverages [20,46,47,48]. Thus, the choice of wood, together with other processing methods, is proving to be a key factor for innovation in the development of alcoholic beverages [7,47,49,50,51,52].

#### 3.2.2. Tropical Woods

In regions where oak cultivation is limited or unfeasible, tropical hardwoods have emerged as promising alternatives, expanding the range of options for crafting innovative sensory profiles. The diversity of tropical species allows for the exploration of unique characteristics, both in terms of chemical composition and cellular structure, enabling the development of beverages with distinctive regional identities and differentiated attributes.

Historically, the use of native woods in Brazil dates back to the colonial period, when the scarcity of oak barrels—due to high import costs and logistical challenges—led producers to experiment with local woods such as *Amburana cearensis* and *Myroxylon peruiferum* (commonly known as amburana and bálsamo, respectively). This process of adaptation and innovation contributed to the development of unique and characteristic sensory profiles in Brazilian cachaça, which today enjoys recognition and appreciation worldwide [9]. The use of native woods represents an opportunity to craft products with strong regional identity while promoting the valorization of Brazilian biomes. Table 1 summarizes the main characteristics and applications of selected tropical woods, emphasizing their potential use in the alcoholic beverage industry.

Amburana wood, characterized by its high porosity, allows for rapid liquid penetration, thereby enhancing the immediate and intense extraction of aromatic compounds. Among the key constituents are coumarin, lactones, and various phenolic acids, which impart distinctive sensory notes such as vanilla, cinnamon, and caramel-like nuances to the beverage. However, this high extraction potential requires strict control over the aging duration to avoid an overwhelming dominance of these aromas, which could unbalance the final sensory profile of the beverage [57,58,59,77,78].

Balsam is another tropical wood that has attracted growing interest. Rich in resinous compounds such as nerolidol, balsam contributes pine- and spice-like aromas, adding a unique and refined aromatic layer to the beverage. Moreover, the phenolic acids present in this wood enhance color stability and contribute to overall sensory complexity, enabling the development of beverages with a well-defined and distinctive character [3,54,55,60,79,80].

Cherry wood (commonly derived from *Prunus serotina*) contains low levels of tannins and high concentrations of aromatic compounds, such as benzaldehyde, vanillin, acetovanillone, and eugenol. These compounds contribute fruity, floral, and sweet notes, making the wood particularly suitable for aging wines, whiskies and liqueurs [51,54,65,66,68,69,74,75,81]. Benzaldehyde provides almond, cherry, and marzipan-like aromas [72,82], while vanillin and eugenol enhance vanilla and spicy nuances [10,24]. The presence of phenolic acids, including gallic and protocatechuic acids, acts both as antioxidants and as precursors to aromatic compounds, in addition to influencing beverage stability [20,36,81]. Furthermore, the flavanol catechin can interact with anthocyanins and tannins, modifying color intensity and astringency in the final product [6,34,67,71,73].

Brazil nut wood, often referred to as “Brazilian oak”, is notable for its high density and low porosity, characteristics that promote gradual maturation and a balanced release of phenolic compounds. Its chemical composition includes gallic and ellagic acids, as well as lactones and aromatic aldehydes, contributing to a complex sensory profile with refined woody notes and subtle spiced undertones, without excessive astringency. This results in a more sophisticated and harmonious beverage [13,57,58,83,84].

In contrast, sweet chestnut, a wood traditionally used in Europe, exhibits greater porosity and a faster gas exchange rate, leading to accelerated aging. This promotes enhanced extraction of ellagitannins and flavonoids, which can intensify astringency [62,81]. While its volatile profile includes furanones and aliphatic phenols (which impart vanilla and coconut aromas), it also tends to release higher concentrations of aggressive compounds such as furans and pyrazines upon toasting [35,64,85]. Therefore, Brazil nut wood emerges as a balanced alternative to European oak, especially when compared to chestnut, providing more controlled extraction, lower astringency, and a gradual sensory evolution that results in a refined and well-integrated product [13,83].

Among other Brazilian hardwoods, *Tabebuia chrysotricha* (commonly known as ipê) is distinguished by its high tannin and lignin content, which significantly influences both the intense coloration and the full-bodied texture of the aged beverage. Its composition includes phenolic acids such as caffeic acid, which contributes to color stability and provides antioxidant properties. The sensory profile is marked by strong woody notes, subtle citrus hints, and nutty aromas that can become more pronounced with increasing toast intensity [60].

*Hymenaea courbaril* (jatoba), on the other hand, is characterized by a high concentration of phenolic acids and vanillin, contributing a yellowish hue and a complex sensory profile. Herbal and mildly sweet notes help balance the flavor of alcoholic beverages, while spicy nuances enrich their character. Native to the Brazilian Amazon and Cerrado biomes, jatoba is a dense wood rich in phenolics that positively influences aging, delivering a persistent and structured aftertaste that is particularly appreciated by those seeking intense flavor profiles [53,54,55,60,79,80,86,87,88].

Jequitiba is known for its sensory neutrality and allows for a gradual release of compounds, which can soften overly intense sensory profiles. Moderate tannin levels reduce astringency, while vanillin and phenolic acids such as syringic acid add subtle vanilla notes and contribute to the smoothness and coloration of the beverage [19,56,57,76].

Peroba wood, due to its high porosity, accelerates the extraction of toasted and spicy compounds. Volatile phenols such as guaiacol and 4-methylguaiacol, along with aromatic aldehydes and furanones, intensify spicy and roasted notes. Additionally, the presence of lactones, gallic acid, and ellagitannins helps to balance sweetness and astringency, enhancing the beverage’s sensory complexity and oxidative stability [13,54,55,57,58,60].

Sassafras (*Sassafras albidum*) wood introduces sweet and spicy notes reminiscent of cinnamon and clove, along with a mentholated touch. Its sensory profile is reminiscent of the characteristic aroma of “root beer”, making it attractive to craft distilleries. However, the presence of safrole—a compound with potential carcinogenicity—has led to strict regulatory limitations regarding its use in food and beverages [16,54,89,90,91]. Other native Brazilian species explored for beverage aging include araribá (*Schinus terebinthifolius*), which is known for a complex aromatic profile with spicy notes and a chocolate-like sweetness [80]. Red cabreúva (*Myrocarpus frondosus*) contributes woody aromas with hints of dried fruit and anise, yielding beverages with a golden hue and full-bodied mouthfeel. Louro-canela (*Ocotea odorifera*), rich in eugenol, imparts cinnamon and clove-like aromas, although its sensory acceptability in cachaça has been reported as negative [19,79,92].

Grapia (*Apuleia leiocarpa*), purple ipê (*Handroanthus impetiginosus*), and cabreúva (*Myrocarpus frondosus*) have shown distinct influences on aged spirits. Grapia resulted in lighter-colored beverages with lower concentrations of phenolic compounds and a natural sweetness. Purple ipê intensified coloration and added aromatic complexity through the presence of syringic acid, vanillic acid, and coniferaldehyde. In this study, cabreúva contributed sweet aromas of vanilla and caramel, associated with vanillin and ellagic acid, and also imparted a velvety mouthfeel [19,76,79,93].

Among tropical species, acacia (*Robinia pseudoacacia*) has been widely studied in Europe for the aging of wines and spirits. It is rich in ellagitannins and volatile compounds such as eugenol, vanillin, and syringol, which impart balsamic, spicy, and vanilla-like aromas. Furthermore, the presence of gallic acid and epicatechin contributes to color development and beverage stability [94].

Temperate-region woods, such as ash (*Fraxinus excelsior*; *F. vulgaris*), exhibit low density and high porosity, releasing lactones, furanones, vanillin, and benzaldehyde. These compounds contribute sweet and spicy notes such as vanilla, coconut, and almond, with a subtle sweetness and spicy undertones when moderately toasted [61,63,70,85].

Additionally, grapevines (*Vitis vinifera*) have been investigated as an alternative aging medium due to their high stilbene content—particularly resveratrol, a phenolic compound known for its health-promoting properties. Resveratrol exhibits strong antioxidant activity and has been associated with the prevention of chronic and degenerative diseases [95]. It also shows anti-inflammatory and antimicrobial effects that may reduce inflammation and fight infection [96]. In addition, resveratrol has been shown to provide protection against cardiovascular disease and may have chemopreventive effects by inhibiting the proliferation of cancer cells [97,98].

#### 3.2.3. Regulatory Aspects of Alternative Woods

The regulatory frameworks governing the use of alternative woods in the aging of alcoholic beverages vary significantly across regions. In the United States, the Alcohol and Tobacco Tax and Trade Bureau (TTB) allows the use of various wood types for aging, provided that products labelled as “straight” are matured exclusively in new charred oak barrels. Although the TTB does not publish a detailed list of authorized alternative woods, it allows their use as long as there is no detectable residue from previous products in the barrels. Regulations also establish specific requirements for categories such as American Single Malt Whiskey, which must be aged for at least two years in oak barrels that meet defined specifications [99].

In the European Union, although community legislation does not provide explicit guidelines regarding alternative woods, the Organisation Internationale de la Vigne et du Vin (OIV) approved the use of oak and chestnut wood chips in the aging of wines in 2005. This was incorporated into European law through Regulations (EC) No. 2165/2005 and No. 1507/2006 [100,101]. Regulation (EU) No. 251/2014 also addresses rules for the production and labelling of aromatized wine products, including aspects related to wood aging [102]. Nevertheless, specific rules on the species of wood and aging methods may vary among EU Member States, as illustrated by Portugal’s Ordinance No. 325/2019, which introduces additional provisions regarding the aging of alcoholic beverages [103].

In tropical regions, where native species such as Amburana are widely available, regulations tend to be more flexible, encouraging sustainable harvesting practices and allowing experimentation with non-traditional woods. In Brazil, the use of wood in the aging of alcoholic beverages is governed by specific regulations, such as Portaria MAPA nº 539 of 26 December 2022, which currently establishes the identity and quality standards for Cachaça and Aguardente de Cana [104].

Producers are advised to carry out preliminary trials to evaluate the sensory contribution and consumer acceptance of beverages aged in alternative woods. Furthermore, the combination of tropical hardwoods with traditional oak can result in balanced and appealing sensory profiles. Collaboration with regulatory bodies is essential to ensure compliance with safety and quality standards. Ensuring sustainable sourcing and educating consumers on the distinctive features of beverages aged in alternative woods may enhance their market acceptance and boost their commercial appeal [20,71,105,106].

### 3.3. Wood Aging Process

The final profile of a wood-aged beverage results from the complex interplay between barrel extractives, environmental conditions, and the intrinsic characteristics of the product itself. These factors collectively define the sensory evolution of the beverage over time. In addition to the species and the geographical origin of the wood, other influential variables include the toasting level, barrel dimensions, usage history, and environmental parameters such as temperature, humidity, and oxygen exposure. These elements modulate the release and transformation of extractable compounds, ultimately shaping distinct sensory profiles and influencing consumer acceptance [107,108,109].

During contact with wood, several phenomena occur that may be broadly classified as follows: additive aging, where wood constituents are extracted into the beverage; subtractive aging, in which compounds are lost through evaporation or absorption; chemical aging, resulting from reactions between beverage components and wood extractives that generate new metabolites; and biological aging, driven by microbial activity that alters the beverage’s composition [44,62,72,109,110,111,112,113,114].

#### 3.3.1. Dynamics of Compound Transfer During Beverage Aging

Throughout the aging process in wooden barrels, compounds are transferred from the wood into the beverage, while water evaporation concentrates volatiles and oxidative reactions promote the formation of new derivatives. This results in color variations, ranging from pale yellow to deep golden hues, and increased aromatic complexity [21,115,116,117,118,119]. Similarly, underwater sea aging has been demonstrated to modify oxygen exposure, enhance compound stability, and increase sensory complexity in red wines [120]. The efficiency of this transfer is determined by wood species and characteristics, the anatomical properties of the barrel’s grain, and usage history: new barrels exhibit greater extractive potential, whereas repeated use leads to the progressive depletion of extractives [24,121,122].

Compound transfer is also modulated by the parameters of the hydroalcoholic solution, including ethanol concentration, temperature, and pH, all of which influence the solubility and hydrophobic behavior of wood constituents. A higher ethanol content enhances the solubility of hydrophobic compounds, facilitating their extraction from the wood matrix [123]. Temperature accelerates diffusion rates and chemical reactions that release extractives, while pH affects the ionization state of phenolic groups, thereby altering their solubility and extraction efficiency [124]. In reused barrels, the wood may release compounds from previously aged beverages, leading to variations in the final profile and contributing to the distinctiveness of products, such as bourbon, whiskey, brandy, and wood-aged beer [24,52,121,123,125,126,127,128].

Furthermore, controlled evaporation and oxidative formation of new chemical compounds enhance the integration of extractives, contributing not only to color development but also to the evolution of the beverage’s sensory attributes. Another aspect to consider during wood aging is the development of acidity and associated microbial activity, both of which may alter compound extraction and significantly influence sensory characteristics. Acidity affects the stability and reactivity of extracted compounds, while microbial activity is shaped by factors such as ethanol concentration and the presence of iso-α-acids. Studies employing synthetic microbial communities have demonstrated that such variables can alter microbial composition, extractive dynamics, and metabolite production, ultimately impacting beverage quality [109].

#### 3.3.2. Origin and Identification of Compounds

The primary compounds responsible for the sensory attributes of wood-aged beverages include volatile terpenoids, phenols, and benzoic and cinnamic aldehydes [36,62]. Many of these compounds originate from the thermal degradation of macromolecules, particularly lignin and hemicellulose, during barrel fabrication, leading to the formation of substances, such as furfural, 5-hydroxymethylfurfural, syringaldehyde, coniferaldehyde, sinapaldehyde, and vanillin. Phenolic compounds, including vanillin and other aromatic aldehydes, influence aroma perception, while furanic compounds affect color, astringency, and bitterness. Beverage quality is often impacted by the extent of wood exposure during maturation, which is strongly related to the beverage matrix, the species and origin of the wood, the maturation period, the surface-to-volume ratio of the barrel, and—where toasted wood is employed—the toasting level [36,62].

Low-molecular-weight phenolic compounds present in wood can be classified as volatile or non-volatile depending on their vapor pressure and chemical nature. Non-volatile phenolics include cinnamic and benzoic acids, flavonols, stilbenes, and other phenolic derivatives, which contribute to color, bitterness, astringency, and beverage stability. Volatile compounds include phenols, aldehydes, alcohols, ketones, furans, lactones, and other aromatic derivatives that contribute to the flavor and aroma of aged beverages. These compounds can be identified using chromatographic techniques coupled with spectroscopic or spectrometric detectors, such as LC-DAD-MS and HS-SBSE-GC/MS, respectively [95].

Several studies have employed advanced instrumental techniques to identify and quantify extractives from alternative tropical woods. For instance, Zacaroni et al. [77] quantified phenolics and coumarins in sugarcane distillates using GC–MS, demonstrating the technique’s effectiveness in characterizing wood-derived extractives. In another approach, Ghanem et al. [89] used peptide receptors combined with multivariate analysis to differentiate wood extracts used in cachaça production, while Martínez-Gil et al. [20] reviewed methodologies for characterizing tannins and low-molecular-weight phenolics, highlighting the importance of chromatographic techniques coupled with spectroscopic detectors (such as LC-DAD-MS and HPLC) for generating detailed chemical profiles. Studies exploring alternative woods, such as those by Castro et al. [57], have also emphasized the utility of traditional methods complemented by spectroscopic approaches like NIR to monitor chemical transformations during aging.

Chemometric approaches and artificial intelligence tools have been increasingly integrated into analytical workflows to facilitate the classification and discrimination of extractives derived from tropical woods. For example, Bortoletto et al. [19] and Bernardes et al. [129,130] developed predictive models correlating phenolic compound concentrations with sensory parameters in distillates aged in non-traditional woods. These methodologies not only improve compound identification and quantification but also enable real-time monitoring of chemical modifications during aging, thus contributing to process optimization and the achievement of desirable sensory profiles.

In addition, certain compounds have been proposed as chemical markers of wood aging. Key markers of aged alcoholic beverages include furans such as furfural and 5-hydroxymethylfurfural, as well as phenolic compounds, like syringaldehyde, coniferaldehyde, sinapaldehyde, vanillic acid, and syringic acid. Research has shown that the concentration of these markers varies according to the wood species used, the aging duration, and storage conditions [87,131]. Examples include trans-resveratrol and vanillin, whose concentration tends to increase during aging, and ellagic acid—a benzoic acid derivative—whose levels decrease over time [95]. Chemical markers specific to oak wood from different origins include cis- and trans-whiskey lactones (associated with coconut notes) and vanillin (associated with vanilla aroma) [28].

In this context, a study by Warren-Vega et al. assessed the quality and authenticity of tequila through a comprehensive review of analytical techniques employed for this purpose. The paper highlights emerging tools supporting the current authentication framework, including the analysis of organic and inorganic markers, spectroscopy, chromatography, and isotopic ratio determination. In addition to examining the effects of barrel aging on tequila, the authors propose analytical parameters to determine the maturation time and regional origin of the product [17].

#### 3.3.3. Evolution of Methods and Techniques for Wood-Aged Alcoholic Beverages

The traditional aging of alcoholic beverages in wooden barrels is still a conventional but costly and time-consuming method. As a result, alternative approaches have emerged that aim to accelerate the aging process by using smaller quantities of wood and different geometries, such as chips, staves, fragments and cubes, to promote more efficient extraction of compounds that affect flavor, aroma and color [46,48,111,132]. Botha et al. [133] showed that, in white wine, oak chips and staves accelerate the release of vanillin and lactones, while full barrels produce a slower yet more integrated extraction profile. Similarly, Tarko et al. [134] demonstrated that aging apple wines with oak chips not only speeds up the liberation of key volatiles like vanillin and furfural but also enhances the sensory parameters of aroma and taste, highlighting the versatility of chip-based aging across different beverages.

Strategies that have been explored to improve the quality of wood-aged alcoholic beverages include techniques such as ultrasound treatments, alternating electrical current, and high-gravity fields [46,135,136]. Nevertheless, one of the most appealing methods from the consumer’s perspective is the use of thermally treated (toasted) wood fragments. This process not only imparts a golden hue to the beverage but also contributes to a smoother mouthfeel and a more balanced perception of ethanol [18,21]. During maturation, a physical alignment of ethanol and water molecules occurs, which reduces pungency and enhances the softness of the final product. Simultaneously, several chemical changes take place, including evaporation, degradation of preexisting compounds, and reactions between beverage constituents and wood-derived compounds. These interactions result in the extraction, absorption, and adsorption of bioactive substances such as flavonoids [64,83,108,122,137,138]. Flavonoids are rapidly oxidized, contributing to the yellowish coloration commonly observed in aged beverages. The application of heat to the wood triggers pyrolysis, which forms pores and channels that enhance both the diffusion of the liquid and the extraction of desirable compounds [44].

Due to the high cost and logistical complexity of barrel usage, alternative aging techniques using wood fragments, such as chips, cubes, staves, or sticks, have gained traction within the beverage industry. These alternatives are more economically viable and environmentally sustainable while also enabling accelerated aging processes [19,37,44,91,109,137]. Despite these innovations, most studies and commercial applications remain focused on oak, a wood species extensively characterized for its chemical and structural properties. However, there remains a substantial knowledge gap regarding alternative wood species, particularly tropical and native species from Brazil, which offer considerable potential for application in aging processes, Amburana being a prominent example.

A study by Coelho et al. [44] examined the reuse of oak chips previously soaked in wine, applied to the aging of beer, wine, and grape pomace spirit (*bagaceira*). The chips had adsorbed both wood-derived and wine-derived volatile compounds, significantly affecting the sensory and physicochemical profiles of the beverages. The wood also acted as a subtractive aging agent. Different formulations were tested using varied concentrations and temperatures, and a stronger perception of woody notes—accompanied by a reduction in fruity and floral aromas—was observed at higher temperatures.

Other research efforts have focused on accelerating the aging of wines [139,140], grape pomace spirits [141], brandies [142,143], and whiskies [144]. Additionally, recent studies have explored the synergy between wood and microorganisms in biological aging processes, particularly in beers [113,145,146,147]. A recent investigation by Tamayo-Sánchez et al. [148] evaluated the color evolution in beverages subjected to accelerated aging with thermally treated French oak chips. Within only four weeks, color profiles similar to those of long-aged beverages were obtained. Light toasting imparted hues ranging from white wine to pale straw and light gold; medium toasting resulted in deeper gold and amber tones; and intense toasting produced oloroso brown and tawny colors. These colorations were associated with the extraction and subsequent oxidation of flavonoids, enabling the construction of a color scale to guide processing decisions and achieve the desired results without the use of additives [148].

Sánchez-Gómez et al. [95] investigated the impact of toasting time and grapevine shoot variety on the composition of low-molecular-weight compounds. Their findings indicated that toasting duration had a more pronounced influence than botanical variety, leading to a decrease in non-volatile compounds and an increase in volatiles. Mild toasting was particularly effective in releasing trans-resveratrol, a bioactive compound recognized for its health-promoting properties.

### 3.4. Impacts of Technological Innovations on Beverage Quality: Recent Research and Applications

In recent decades, research on the wood aging of alcoholic beverages has advanced well beyond the exclusive use of oak, incorporating new wood species and techniques that enable more precise and sustainable modulation of sensory profiles. Strategies, such as microbial consortia [113,114], high-resolution metabolomics [124], high-resolution spectrometry for the characterization of phenolics and volatiles [83,138], intelligent barrel reuse [149], sorption modeling [123] and accelerated aging by ultrasound and electric field [136,137,143,150], have all expanded the use of alternative woods in wines, beers, cachaças, and other spirits. Meanwhile, controlled-toast wood fragments [13,44,52,61,62,63,67,68,74,95,151,152], flavored lees [70] and targeted microbial inoculation [109,113] offer the directed release of aroma compounds.

To systematize these efforts, Table 2 presents a thematic overview of studies organized into four principal axes: Aging Materials and Systems, Analytical Techniques and Classification Tools, Chemical Composition and Interactions, and Contextual and Market Perspectives. Each axis details the specific aspect investigated, the beverage type(s), and the corresponding references, thus providing a clear visualization of applied innovations (storage conditions, native or alternative woods, energetic and biotechnological accelerations) of the analytical tools employed and of the sensory, chemical, and market impacts observed. This structure facilitates the identification of knowledge gaps and guides future advances in the quality, authenticity, and sustainability of wood-aged alcoholic beverages.

Using advanced techniques, such as SPME-GC–MS, HPLC-DAD, DTD-GC–MS and high-resolution spectrometry, numerous studies have elucidated the mechanisms of volatile and phenolic compound extraction from wood during spirit maturation. Coldea et al. [151] compared aroma profiles in pálinka and tequila aged in different wood essences, highlighting how wood choice can enhance specific fruit and spice notes. In cachaça, Barbosa et al. [83] quantified phenolic compounds and lignin degradation markers, while Castro et al. [138] investigated quality indicators, including soluble lignin fractions, in oak barrels of different provenances. Complementarily, Santiago et al. [54,60,78,167] and Zacaroni et al. [77,92] studied cachaças matured in native woods (amburana, jatobá, balsam, and peroba), showing how wood porosity and chemical composition modulate the volatile profile and congener development over time. In cane- and agave-based spirits, Aguilar-Méndez et al. [21] traced volatile compound evolution in tequila aged in regenerated barrels, and Bortoletto et al. [19] monitored phenolic transformations in cane spirit using various tropical fragments. Lima et al. [55] provided an overview of phenolics in sugarcane spirits and related aging parameters to quality control procedures, while Fernandes et al. [168] used high-resolution spectrometry to identify gallotannins and ellagitannins in alternative aging methods, demonstrating the power of such approaches to detect difficult-to-detect phytochemicals.

Moreover, several authors have mapped the chemical composition and assessed the sensory impact of alternative woods used in beverage aging, underscoring the diversity of species with enological and technological potential. Híc et al. [2] evaluated the use of multi-species fragments in spirit maturation, highlighting variability in extractable compounds and their influence on the beverage profile. In a more historical and comprehensive approach, Zamora [24] revisited the use of different wood types over time, reflecting on their implications for the final quality of aged products. Comparative studies such as Jordão et al. [69] revealed significant differences in the volatile and phenolic profiles of beverages matured in cherry, acacia, and oak, underscoring the importance of botanical selection for targeted sensory outcomes.

In the context of cachaça, Catão et al. [80], Bortoletto and Alcarde [87] and Briceno et al. [177] advanced the chemical quality assessment of spirits aged in Brazilian tropical woods by quantifying key congeners and impact compounds, while Castro et al. [57] extended this scope to include Amazonian species. Zacaroni et al. [77] further characterized phenolic compounds and coumarins in cachaças matured in different woods, highlighting the phytochemical diversity of native species. Comparative studies on traditionally used European woods have also provided valuable insights: Canas et al. [64] examined chemical and sensory differences between oak and chestnut in wine–brandy maturation, and Bargalló-Guinjoan et al. [106] evaluated Mediterranean species in accelerated-aging protocols, proposing viable industrial alternatives. García-Moreno et al. [72] investigated the aging of Sherry Oloroso in four distinct woods, revealing compositional variations that directly influence final product quality, and Warren-Vega et al. [115] detailed how barrel properties modulate the chemical composition of tequila. In brewing applications, Wyler et al. [147] assessed the impact of oak barrel aging on beer sensory profiles, whereas Correia et al. [164] and Cioch-Skoneczny et al. [170] explored short-term macerations with wood chips to induce rapid aroma and flavor changes. Additionally, Smailagić et al. [73] proposed analytical methodologies to discriminate wood species based on controlled compound extraction, contributing to traceability tools. Within oenology, Martínez-Gil et al. [5,75] deepened the volatile and phenolic characterization of alternative cooperage materials, such as *Quercus humboldtii*, in comparison to traditional oaks, highlighting the potential of under-explored species to diversify wine flavor profiles. Complementarily, Simón et al. [61] identified species-specific polyphenols among different oaks, which improves our understanding of the chemical determinants of sensory quality in barrel-aged wines.

The intrinsic aroma of the wood itself has also been systematically mapped: Ghadiriasli et al. [3,28] employed comprehensive two-dimensional GC–MS coupled with olfactometry to identify terpenes and lactones (e.g., β-methyl-γ-octalactone, guaiacol) in oaks from various origins. Guerrero-Chanivet et al. [163] demonstrated that traditional “seasoning” of Sherry casks suppresses native oak lactones while promoting sotolon and furfural accumulation, underscoring the critical role of barrel pre-treatment. Similarly, Garrido-Bañuelos and Buica [178] identified a strong correlation between the presence of furanmethanethiol (FMT)—a compound associated with toasted oak precursors like furfural—and the perception of coffee aroma in Pinotage wines, reinforcing the sensory impact of thermally derived wood volatiles. Jordão and Cosme [10] modeled wine sorption kinetics across different wood species, and Coelho et al. [123] described in detail the absorption and retention kinetics of volatiles by oak, reinforcing its function as an active sorptive matrix. Briceno et al. [177] applied Peleg’s kinetic model to cachaça aged in amburana, balsam, Brazil nut, and oak, demonstrating a higher initial production rate of esters and aldehydes in tropical woods. Over 48 months, low-molecular-weight volatiles initially predominated, while phenols and ethyl palmitate became dominant in later stages, shaping the beverage’s sensory evolution. To optimize phenolic extraction, Trillo-Ollero et al. [172] applied kinetic models in Brandy de Jerez that correlate wood-to-wine surface area ratios with tannin release and oxidation rates, thus providing predictive criteria for barrel design. Similarly, Carpena et al. [37] investigated the effect of wood porosity, micro-oxygenation, and barrel geometry on wine maturation, and López-Solís et al. [149] validated the reuse of barrels in Carménère aging, showing regenerated casks maintain phenolic profiles comparable to new ones. Coelho et al. [44] also highlighted the sustainable potential of recycled oak staves to modulate volatile composition and promoted environmental aging strategies.

Finally, toast level and fragment type prove to be important levers for sensory tuning. Sánchez-Gómez et al. [95] observed significant shifts in low-molecular-weight phenolics upon toasting vine shoots, while Coelho et al. [122] established a relationship between ethanol concentration and temperature to volatile extraction in oak chips. Bajerski et al. [11] showed that toasted oak chips markedly alter beer aroma, and Baiano et al. [14] demonstrated in Aglianico and Montepulciano wines that medium toasting maximizes fruity esters without compromising color. Navarro et al. [52] compared French versus American oak, revealing toast-level-dependent ellagitannin release, and Pichler et al. [65] found heavy toasting accentuates smoky notes in Merlot, whereas lighter toasting preserves fresh fruit character. Studies by Chira and Teissedre [152] and González-Centeno et al. [169] confirmed that both oak provenance and toast intensity define distinct volatile and tannin signatures, and Rodríguez-Solana et al. [141] employed a Box–Behnken design to optimize grape-marc fragment size and toast level for accelerated aging. Broadening this scope, Híc et al. [2] and Correia et al. [164] evaluated tropical and cherry wood fragments, respectively, demonstrating the rich array of approaches available for sensory diversification.

In recent years, various physical and energy-based approaches have been proposed to drastically shorten maturation times without compromising the final sensory profile. Zeng et al. [136] demonstrated that applying an alternating electric field significantly alters the kinetics of phenolic extraction in wine, achieving maturation levels comparable to traditional barrels in a fraction of the time. Complementarily, Schwarz et al. [143] and Abreu-Naranjo et al. [137] validated ultrasound-assisted protocols—both alone and in combination with oak chips—to accelerate the aging of brandy and sugarcane spirit, respectively, obtaining volatile and sensory profiles analogous to those from long-term maturation. Jiménez-Sánchez et al. [150,154] extended these techniques to Jerez vinegar at pilot scale and wine, confirming their sensory viability. Conner [18] established the theoretical framework underpinning these accelerated-aging technologies, while recent reviews by Pizarro et al. [132], Tao et al. [139], Pielech-Przybylska and Balcerek [144], Bossaert et al. [153], Krüger et al. [155], and Wang et al. [175] discussed integrated strategies—pressurization, controlled heating, and AI-guided micro-oxygenation—to optimize the extraction of aromatic and phenolic compounds without resorting to prolonged barrel times.

Concurrently, biotechnological approaches have been explored to mimic or shorten barrel contact while preserving the characteristic sensory complexity of barrel aging. Coelho et al. [113] demonstrated that co-fermentations of *Saccharomyces cerevisiae* and *Dekkera bruxellensis* in beer can reproduce, within weeks, volatile transformations typically associated with years of barrel maturation. Snauwaert et al. [114] characterized microbial community dynamics in Belgian acid ales, showing how native bacteria and yeasts control the production of fruity and sour notes, while Bossaert et al. [1,9,108] advanced this understanding by monitoring microbial succession and the influence of ethanol and iso-α-acid levels on final beer chemistry during wood maturation. In distilled spirits, Caetano et al. [165] applied Kohonen neural networks to correlate microbial metabolite profiles with sensory perceptions in cachaça, paving the way for data-driven fermentations. Finally, Palomero et al. [70] employed the impregnation of yeast lees with extracts from various woods, demonstrating that phenolic and volatile compounds adsorbed to cell walls are released in a controlled manner during red wine fermentation and maturation, thus partially decoupling aging from direct barrel contact.

Beverage coloration evolves dynamically during aging and can be finely tuned by toast level, oxygenation, and selective colorants. Delgado-Gonzalez et al. [173] and Warren-Vega et al. [161] developed kinetic models to quantify chromatic changes in distilled spirits and tequilas, accounting for both wood type and aging duration. In tequilas, electrochemical color indices coupled with machine learning algorithms enable the precise discrimination of variety and age [17,162], while Tamayo-Sánchez et al. [148] showed that thermally treated oak chips can impart barrel-like hues in just four weeks. Concurrently, Ríos-Hernández et al. [174] designed tailor-made charcoals to selectively remove color from Cristalino tequila without compromising its sensory profile. In wine brandy, Canas et al. [64,142] demonstrated that controlled micro-oxygenation governs not only initial color intensity but also phenolic pigment fixation. For red varieties such as Sangiovese, Gambuti et al. [171] found that calibrated oxygen doses promote tannin–anthocyanin polymerization, yielding more stable coloration. Coetzee and du Toit [176] reviewed how oxygenation in Sauvignon Blanc enhances thiol aromas and prevents faults. Finally, Santiago et al. [54,167] quantified pH, total tannin, and color variations in cachaças aged in diverse woods, reaffirming the influence of barrel material on final appearance.

The need to reduce the impact on the environment has driven the introduction of circular economy practices in cooperation. Reused barrels and oak chips have been shown to retain their extraction capacity and sensory quality, making their reuse both economically and environmentally beneficial [44,126,127]. Rapid assays for detecting barrel reuse markers in cachaça further support effective monitoring of these sustainable practices [131], while production-chain mapping highlights criteria for selecting and refurbishing barrels under stringent sustainability standards [166].

Ensuring the provenance and integrity of wood-aged beverages has spurred the development of advanced analytical tools. Peptide-based receptors and multivariate analysis can generate unique wood “fingerprints” [89] and chemometric models discriminate commercial cachaças by their chemical profiles [129]. Machine learning classifiers have been successfully applied to identify wood species in aged spirits [156], while in brewing, volatile-fingerprinting assays estimate aging time in beer [106]. Ziyatdinova et al. [159] and Sánchez-Guillén et al. [160] used electrochemical techniques to authenticity in brandies and other spirits and to assess antioxidant activity. Complementary spectroscopic and metabolomic methods, including FT-IR for wine [116], isotopic ratios for tequila [115], and FT-ICR-MS for whisky [124], complete a robust toolkit for quality assurance and authenticity verification.

## 4. Future Perspectives

The aging of alcoholic beverages in wooden barrels is a well-established practice that significantly contributes to the sensory complexity of products such as whiskey, wine, tequila, and beer. Traditionally dominated by the use of European and American oak, this process has been expanded through research focused on innovative alternatives, aiming to diversify sensory profiles and promote greater sustainability.

Current research focuses not only on the characterization of non-traditional woods but also on the development of new techniques, such as the use of wood fragments, thermal treatments, and emerging technologies, including ultrasound and alternating current. These methods have shown potential to accelerate the aging process and modulate wood–beverage interactions, paving the way for promising discoveries in the production of differentiated, customized, and premium beverages.

However, the use of alternative woods still presents significant challenges, particularly regarding a detailed understanding of their physical and structural properties. While oak’s anatomical and chemical characteristics are extensively studied, such as the presence of tyloses, density, pore size, and cell wall composition, many alternative species still lack in-depth research. Cell structure has a direct influence on the rate of micro-oxygenation and the release of phenolic and aromatic compounds, key elements that determine the final product’s sensory quality.

Moreover, most studies on alternative woods focus on short aging periods. Long-term research is needed to evaluate color stability, the evolution of volatile and phenolic compounds, and ester formation over time. Brazilian species, such as amburana, jatoba, and ipê, for example, show significant sensory potential, but gaps still remain in correlating their microstructure with the sensory effects observed during extended aging.

Advancing knowledge in this area depends on the integrated application of techniques such as electron microscopy, mass spectrometry, gas and liquid chromatography, along with sensory evaluations by trained panels. Furthermore, statistical modeling and the use of artificial intelligence may be useful strategies to predict the sensory behavior of beverages based on the characteristics of the wood and aging conditions, optimizing the process and ensuring consistency.

The valorization of alternative woods to oak, when associated with sustainable forest management practices, represents a concrete opportunity for innovation. This approach contributes to the preservation of natural resources, stimulates regional production chains, and enables the development of beverages with unique cultural identity. The dialogue between science, technology and tradition is, therefore, crucial for broadening the horizon of aged beverage production and creating a more diverse, sustainable and sensorially richer sector.

## Figures and Tables

**Table 1:** Characteristics and applications of tropical wood types in aged alcoholic beverages: evidence from studies published between 2014 and 2024.

Wood Type	Scientific Name	Applications	Key Characteristics and Sensory Profile	References
Amburana	*Amburana* *cearensis*	Cachaça, rum, brandies	Durable and moderately extractive wood. Rich in coumarin, lactones, gallic acid, and other phenolic compounds. Imparts smooth vanilla, almond, sweet spice, coconut, and dried fruit notes.	[13,53,54,55,56,57,58,59,60]
Balsam	*Myroxylon* *peruiferum*	Cachaça, rum, other spirits	Soft and easy to handle, less durable. Contains gallic acid, ellagic acid, vanillin, eugenol, and intense aromatic compounds. Produces resinous notes with pine, vanilla, and spices.	[3,13,54,55,57,58,59]
Brazil Nut	*Bertholletia* *excelsa*	Brandies, cachaça, other spirits	Extremely dense and heavy, with low porosity and gradual compound release. Rich in phenolic compounds, lactones, and aromatic aldehydes. Yields an elegant profile with woody, spicy notes and subtle dried fruit nuances.	[13,54,57,58]
Chestnut	*Castanea* *sativa*	Brandies, cachaça, wines, other spirits	Highly porous with good extractive capacity and robust structure. Contains ellagic acid, ellagitannins, flavonoids, furanones, and volatiles like furanes and guaiacol. Delivers toasted, smoky, spicy aromas, with vanilla, coconut, caramel, and spice notes. Higher astringency with antioxidant properties.	[61,62,63,64]
Cherry	*Prunus avium*, *Prunus* *serotina*	Brandies, liqueurs, whiskies, wines	Moderately structured wood with low tannin levels. Rich in benzaldehyde, vanillin, acetovanillone, eugenol, gallic acid, and catechin. Produces fruity, sweet, and slightly floral notes with almond undertones.	[6,10,20,24,51,54,56,61,63,65,66,67,68,69,70,71,72,73,74,75]
Ipê	*Tabebuia chrysotricha*, *Handroanthus* spp.	Cachaça, wine, other spirits	Very dense and resistant with gradual compound release. Contains tannins, lignins, and phenolic acids such as caffeic acid. Produces intense woody and spicy notes, orange hue, smooth mouthfeel with slight astringency.	[19,53,55,57,58,76]
Jatoba	*Hymenaea* *courbaril*	Brandies, cachaça	Dense, robust, and aromatic wood. Contains phenolic acids and vanillin, with a profile similar to oak. Provides herbal, woody aromas, balanced sweetness/acidity, and a hint of bitterness.	[13,53,54,55,56,57,58,60]
Jequitiba	*Cariniana* spp.	Cachaça, other spirits	Wood with moderate porosity and extractive capacity, rich in coumarins and phenolic acids. Imparts spicy, woody, and vanilla notes, with a smooth aging evolution.	[19,57,76]
Peroba	*Paratecoma peroba*	Cachaça, brandies, other spirits	Wood of moderate density and medium porosity, rich in coumarins, vanillin, and phenolic compounds. Confers sweet, floral, and spicy notes with hints of honey, vanilla, and a balanced woody character.	[13,54,55,57,58]

**Table 2:** Thematic overview of scientific publications on wood-aged alcoholic beverages.

Main Topic	Aspect Investigated	Beverage(s)	References
Aging Materials and Systems	Aging Conditions and variable influence	Beer	[147,153]
		Brandy	[143]
		Spirits	[18,154]
		Wine	[7,71,122,127]
	Technological strategies for aging	Alcoholic beverages	[3,28,68,155]
		Beer	[113]
		Brandy	[156]
		Cachaça	[115,157]
		Spirits	[137,141]
		Wine	[70]
		Wine spirits	[142]
	Traditional, alternative, and native wood species	Alcoholic beverages	[44,95,105,148]
		Brandy	[62]
		Cachaça	[19,54,78,87,89]
		Spirits	[2,57,73,158]
		Wine	[5,24,61,63,69,72]
Analytical Techniques and Classification Tools	Analytical and instrumental methods for assessing aging effects	Alcoholic beverages	[3,28,68]
		Beer	[106]
		Brandy	[159,160]
		Cachaça	[92,130]
		Tequila	[17,21,161]
	Evaluation Methods and Analytical Approaches	Tequila	[152,162,163]
	Sensory Outcomes and Wood-Influenced Quality	Beer	[164,165]
		Brandy	[74]
		Tequila	[151]
		Wine	[14,56,63,132]
	Analytical Techniques and Classification Tools	Cachaça	[129,157]
		Tequila	[117]
		Whiskey	[124]
		Wine	[116]
Chemical Composition and Interactions	Major compound groups	Beer	[11]
		Cachaça	[55,60,138,166,167]
		Spirits	[151]
		Wine spirits	[168]
		Wine	[6,52,65,169]
	Wood–beverage interactions	Alcoholic beverages	[58,64]
		Beer	[13,170]
		Cachaça	[83]
		Tequila	[115]
		Wine	[10,123,149,171]
	Microbiology and Biochemical Processes	Beer	[108,109,114]
	Chemical transformations during aging	Brandy	[111,172]
		Spirits	[173]
		Tequila	[174]
Contextual and Market Perspectives	Literature reviews on aging materials and methods	Alcoholic beverages	[121,155,175]
		Wine	[37,75,139,176]
	Emerging trends and consumer preferences in aged alcoholic beverages	Alcoholic beverages	[144]

## Data Availability

The original contributions presented in this study are included in the article; further inquiries can be directed to the corresponding author.
